# Longitudinal Cohort Study of the Relationship Between Illness Perception, Perceived Social Support, and Psychosocial Quality of Life in Adolescents and Young Adults Newly Diagnosed with Cancer: Outcomes from a BRIGHTLIGHT Study

**DOI:** 10.3390/cancers17121918

**Published:** 2025-06-09

**Authors:** Bethany Wickramasinghe, Lorna A. Fern, Rachel M. Taylor, Richard G. Feltbower

**Affiliations:** 1 Department of Targeted Intervention, University College London, London WC1E 6BT, UK; bethany.wickramasinghe.19@alumni.ucl.ac.uk; 2 Cancer Clinical Trials Unit, University College London Hospitals NHS Foundation Trust, London NW1 2PG, UK; lorna.fern@nhs.net; 3 Centre for Nurse, Midwife and Allied Health Profession Research (CNMAR), University College London Hospitals NHS Foundation Trust, London NW1 2PG, UK; 4 Leeds Institute for Data Analytics, School of Medicine, University of Leeds, Leeds LS2 9JT, UK; r.g.feltbower@leeds.ac.uk

**Keywords:** adolescent, young adult, cancer, social support, peer support, illness perception, BRIGHTLIGHT, psychosocial quality of life

## Abstract

Background/objectives: Social support can enhance psychosocial health-related quality of life (PSQOL) in adult cancer patients. Adolescents and young adults (AYAs) with cancer face unique psychosocial challenges that intersect with key developmental milestones. Theoretical models propose that illness perceptions and social support are key determinants of coping strategies and long-term health outcomes in this context. These may be especially salient for AYAs, for whom peer relationships and identity formation are central. Methods: We explored how perceived social support and illness perceptions influence PSQOL over time in AYA cancer patients through a secondary analysis of the BRIGHTLIGHT longitudinal cohort study. Results: BRIGHTLIGHT followed 830 young people aged 13–24 across five time points (6–36 months post-diagnosis). Multi-level modelling revealed that PSQOL improved over time but remained consistently lower in females (mean: 69.62, 95% CI: 70.69 to −68.55). Greater perceived support from friends was associated with poorer PSQOL (β: −0.77, 95% CI: −1.007 to −0.54) and linked to negative illness perceptions, longer hospital stays (β: 0.01, 95% CI: 0.00 to −0.02), longer diagnostic intervals (β: −0.009, 95% CI: −0.02 to −0.00), and poorer clinical communication (β: 0.52, 95% CI: 0.01 to −1.03). A patient interpretation exercise with BRIGHTLIGHT’s Young Advisory Panel contextualized these findings. Conclusions: While peer support could promote normalcy, it could also intensify distress through emotional pressure or social isolation. Future research should address not only access to social support but its quality and relevance to AYAs’ unique psychosocial needs.

## Simple Summary

Adolescents and young adults with cancer often face emotional and social challenges, distinct from those experienced by older adults. We explored how young people’s understanding of their cancer and the social support they receive can affect their psychosocial wellbeing over time. Using data from a large national study, we followed cancer patients aged 13 to 24 for three years after diagnosis. While overall wellbeing improved, it remained lower for females. Greater social support from friends was linked to poorer psychosocial wellbeing and perceptions about cancer. A patient interpretation exercise showed this was often due to feeling overwhelmed, isolated, or misunderstood. Social support from friends plays a unique role in mediating psychosocial wellbeing in this population, as it can help maintain normalcy. However, its impact depends on timing and quality. Support systems must be adapted to meet the specific needs of this group, guiding both service provision and future research.

## 1. Introduction

Adolescents and young adults (AYAs) with cancer are defined in the United Kingdom (UK) as those aged 16–24 [1]. Each year, approximately 2400 new cancer diagnoses are made in this group [2]. Compared to younger children and older adults, AYAs are at a critical developmental period of identity formation, social development, and education [3]. Although disruptive at any age, a cancer diagnosis during this life phase has an acute psychosocial impact [4,5]. Beyond the immediate physical burden, AYAs face disruptions in self-image, personal identity, and relationship dynamics [6,7,8,9]. Cancer and its treatments can interfere with key social milestones such as graduating from school, establishing peer and romantic relationships, and planning for future careers [10,11]. As a result, many AYAs experience social isolation, which adversely affects self-esteem and overall wellbeing [12,13,14]. Moreover, long-term effects (such as cognitive impairments, fatigue, and persistent anxiety) often continue well after treatment, complicating efforts to reintegrate into everyday life [15,16,17].

From a health system’s perspective, AYAs fall between paediatric (with an average age of 6) and adult (with an average age of 66) oncology services [18]. Consequently, AYAs often find themselves in a “no man’s land”, where age-appropriate support is limited [19]. Many AYAs experience a lack of autonomy, peer connection, and support to complete developmental milestones [20,21]. Appropriate evidence to support ongoing improvement and development of targeted interventions is needed to support AYA cancer patients throughout their treatment and survivorship journey.

### 1.1. Theoretical Models of Illness Perception and Social Support

Illness perception refers to how individuals understand and interpret their illness [22]. According to Leventhal’s model of illness representation and regulation, these perceptions influence coping strategies and overall health outcomes [23]. Illness perceptions during treatment are associated with fear of recurrence in child and adult survivor populations [24,25]. In adult cancer patients, negative illness perceptions are often associated with poorer psychosocial outcomes [25,26]. Although research with AYAs is less extensive, studies indicate that cancer challenges AYAs’ developing identity and that social engagement with peers plays a key role in this process [13,27].

Parallel to illness perceptions, psychosocial models of disease highlight the importance of social support in influencing general wellbeing and health outcomes [28,29,30,31,32]. The role of social support in aiding adjustment to cancer through bolstered physical, psychological, and emotional adjustment in adult cancer patients is widely documented [33,34]. Greater social integration is frequently associated with better cancer survival and quality of life in these patients [35,36], which can facilitate psychological adaptation and physiological healing following treatment [37]. Similar benefits have been observed among AYAs, including decreased psychological distress [38], reduced illness-related uncertainty [27], improved coping [39], and increased quality of life [14]. However, despite these benefits, young people with cancer still experience challenges with social relationships and difficulty achieving expected social outcomes [7]. The psychosocial needs of AYAs with cancer remain largely unmet, indicating the need for a more nuanced understanding of the developmental context of this population [10,40].

### 1.2. Methodological Challenges of AYA Research

However, conducting research with young people with cancer presents several methodological challenges. Firstly, the diversity within the AYA age range [1] often results in inconsistent definitions. Secondly, the unique combination of physical, emotional, and social challenges faced by young people complicates the assessment of psychosocial outcomes, often resulting in a lack of validated measures for this group. Thirdly, recruitment is further complicated by the transitional nature of this life stage and variable health statuses, leading to small sample sizes and predominantly cross-sectional study designs [10,41]. These factors limit the generalizability of existing findings. Our study sought to address these gaps using a theory-informed approach that integrated longitudinal quantitative analysis with patient interpretation. Guided by models of illness perception and social support, we aimed to investigate how these factors influence the psychosocial needs and quality of life of adolescents and young adults (AYAs) with cancer.

## 2. Methods

### 2.1. Study Design

We conducted a secondary quantitative analysis of the BRIGHTLIGHT dataset [42] followed by a patient interpretation exercise to contextualize our findings. The BRIGHTLIGHT cohort was and remains the largest longitudinal cohort study of AYAs globally. The comprehensive dataset includes patient-reported clinical and nationally derived data from the National Health Service (NHS) repository. The patient interpretation exercise involved BRIGHTLIGHT’s Young Advisory Panel (YAP) to contextualize and inform the interpretation of the quantitative findings.

BRIGHTLIGHT was a longitudinal cohort study conducted in England by obtaining data from AYAs through a bespoke survey [43]. The survey contained five validated questionnaires and 169 descriptive questions related to pre-diagnostic and post-diagnosis experience. These provide comprehensive information on psychosocial factors, clinical characteristics, and survivorship outcomes.

### 2.2. Participants

#### 2.2.1. Quantitative Study

The BRIGHTLIGHT survey was administered at five time points during the first 3 years after diagnosis (6, 12, 18, 24, and 36 months that corresponded to data collection waves 1 to 5, respectively). Details of recruitment and the cohort are reported previously in detail [42,44]. The BRIGHTLIGHT study sample comprised AYA cancer patients recruited from 97 hospitals across England who were diagnosed between 1 July 2012–31 December 2014. Young people were eligible for inclusion if they had a new cancer diagnosis and were aged 13–24 years at the time of diagnosis. The only exclusion criteria were those who were at the end of life, incarcerated, or incapable of completing a survey. The age of inclusion reflects the criteria for service delivery at the time of recruitment, with services that had adolescent units admitting young people aged 13–24, while those with AYA services admitting those aged 16–24.

#### 2.2.2. Patient Interpretation Exercise

The YAP had been involved in BRIGHTLIGHT as co-researchers since the study’s inception. They named the study and contributed to the development of its overall design, its research questions and outcome measures, dissemination of results, and identification of future research directions [45]. Active members of the YAP were invited to participate in a focus group discussion to contextualize the quantitative findings through their own cancer experiences.

### 2.3. Quantitative Study

#### 2.3.1. Data Collection

BRIGHTLIGHT data were collected from three sources: (1) young people’s self-report via the BRIGHTLIGHT survey [43], (2) patient clinical records, and (3) cancer registry data from National Cancer Registration and Analysis Service (NCRAS) databases. Data collection occurred multiple times along the cancer journey, including following diagnosis, during treatment, and in post-treatment/survivorship phases. The BRIGHTLIGHT survey was administered through face-to-face interviews in young people’s homes by an independent research company at wave 1. Subsequent waves of data collection took place either online or by telephone interview.

As part of the BRIGHTLIGHT survey, participants completed standardized questionnaires to assess psychosocial health-related quality of life (PSQOL), social support (MSPSS), and illness representation (BIPQ, Table 1). The MSPSS distinguishes perceived social support from friends, family, and a significant other but does not specifically capture whether ‘friends’ had a lived experience of cancer. In our study, ‘friends’ predominantly referred to pre-existing social contacts without a cancer diagnosis. At the time of study development (2011), the PedsQL was the most appropriate measure of PSQOL validated for AYAs [43].

Data from NCRAS databases included the date of diagnosis and treatment data, which were used to supplement and validate details of treatment received. Demographic and clinical information, including cancer type, treatment regimens, and disease stage, was also collected from medical records.

#### 2.3.2. Analysis

Descriptive statistics were used to summarize the demographic and clinical characteristics of the BRIGHTLIGHT cohort as well as longitudinal trends in PSQOL, social support, and illness perception.

Three separate mixed effects multivariable regression models were used to assess (1) the independent contributions of perceived social support and illness representation to PSQOL over the 3 years since diagnosis, (2) the contributory factors related to perceived social support from friends, and (3) the contributory factors related to the overall impact of cancer on participants’ lives (domain 1 of the BIPQ).

Each model was optimized to ensure goodness of fit using the Bayesian Information Criterion (BIC) and incorporated random slopes and two-level variance components (with person at level two) to account for repeated measurements taken over the study period. Each model included a minimal adjustment set of confounders identified using a Directed Acyclic Graph (DAG) based on a causal inference approach [49] (DAGitty software: www.dagitty.net, Supplemental Figure S1). Potential confounding variables selected for inclusion in the DAG were identified using prior literature [50]. The pool of potential confounding variables included age at diagnosis, gender, cancer type (haematological and solid tumours), socioeconomic status (Index of Multiple Deprivation quintile based on postcode at diagnosis), cancer severity (a bespoke metric: least, intermediate, and most) [42], ethnicity (white and other), presence of any long-term condition prior to cancer (yes/no), days from first symptom to diagnosis, number of general practitioner visits before diagnosis, treatment group (combinations of chemotherapy, radiotherapy, surgery, or transplant), geographical location (specified as 12 cities, derived from the specialist AYA unit and their network of hospitals), and communication from cancer specialists and nurses. The final minimum adjustment sets for each model are detailed in the tables the models are presented.

Interaction terms between age and gender were tested in model 2. There was no evidence for an interaction effect in models 1 and 3 based on the low Bayesian Information Criterion (BIC, Supplemental Table S2). An analysis was conducted using STATA version 18.

### 2.4. Patient Interpretation Exercise

#### 2.4.1. Data Collection

The research team facilitated an online 2-h focus group with the YAP via Zoom (chosen by the YAP as their preferred platform) under a secure subscription account. The session was digitally recorded and transcribed verbatim using Otter.ai. Its aims were to present the key study findings to the YAP, examine how these related to the YAP’s own cancer experiences, and explore insights for future research and service development.

The focus group comprised a slide presentation of the key study findings followed by a semi-structured focus group discussion. The discussion was divided into three thematic sections based on the quantitative study results (Supplemental Figure S2 and Table S1). During the discussion, YAP members were invited one by one to share their insights and reflect on how the results aligned with their own experiences. Questions such as “Does this make sense to you and why?” and “How does this relate to your experience” were posed to stimulate thought and facilitate participation. Key prompt questions related to each discussion topic were shared with the YAP in advance of the meeting, alongside an information sheet, agendas, consent forms, and a written summary of the study results. This allowed time to reflect and potentially prepare ideas before coming together.

#### 2.4.2. Analysis

An adaptation of framework analysis methods [51] guided our approach to organizing, interpreting, and synthesizing the qualitative data in relation to the key findings from the quantitative study.

Two members of the research team independently verified the transcript against the original audio recordings to ensure accuracy and establish a clear understanding of the dataset before progressing with analysis. Data were initially categorized into descriptive groupings based on the quantitative findings and the discussion guide (e.g., “gender differences in PSQOL”, “helpful social support”). These initial groupings served as the analytical framework for subsequent analysis steps. The transcript was then imported into NVIVO14 for inductive coding by the lead researcher. This involved segmenting the text into meaningful fragments that were grouped according to recurrent and salient categories.

Codes were mapped onto the analytical framework to generate a comprehensive codebook and subsequently exported to Excel to create a coding matrix. This process helped to establish a cohesive narrative across both datasets. Salient participant quotes were extracted to enrich the narrative, and team discussions ensured that the final themes accurately reflected participants’ interpretations of the results.

## 3. Results

### 3.1. Quantitative Study

#### 3.1.1. Demographics

Demographic characteristics are summarized in Table 2. Linked BRIGHTLIGHT survey data at wave 1 were available for 830 participants.

#### 3.1.2. Longitudinal Changes

PSQOL scores initially increased rapidly between waves 1 and 2 (mean scores between waves 1 and 2: 71–76, median scores between waves 1 and 2: 72–78) before plateauing. Social support scores followed a similar upward trend over time between waves 1 and 2 (mean scores for perceived social support from friends: 8–10, median scores for perceived social support from friends: 7–8) that eventually plateaued over the study period. Considering the different social support domains, perceived social support from friends was consistently highest across the study period compared to those for support from family or a significant other. Marginal gender differences were observed between the different domains of social support over time. Negative illness perceptions (consequences, identity, coherence, emotional representation, and concern domains) decreased over time, but positive illness perceptions (personal and treatment control domains) increased over time (Table 3).

#### 3.1.3. The Independent Contributions of Perceived Social Support and Illness Representation to PSQOL Outcomes over the 3 Years Since Diagnosis

Model 1 (Table 4) showed that PSQOL scores were consistently higher in male participants across the study period (mean: 79.18, 95%CI: 80.10 to −78.27) compared with female participants (mean: 69.62, 95% CI: 70.69 to −68.55). Greater social support from friends was related to lower PSQOL scores (β: −0.77, 95% CI: −1.007 to −0.54). Longer diagnostic intervals and time spent in hospital during the first 12 months since diagnosis were associated with lower PSQOL (β: −0.009, 95%CI: −0.02 to −0.00). Similarly, a perception of a greater overall impact of cancer (β: −0.94, 95% CI: −1.35 to −0.53), more side effects (β: −0.75, 95% CI: −1.14 to −0.37), greater concerns about cancer (β: −0.37, 95% CI: −0.80 to −0.07), and greater emotional impact of cancer (β: −1.56, 95% CI: −2.02 to −1.11) were related to poorer PSQOL scores. However, the greater the extent to which participants believed that their treatment can or had helped was related to higher PSQOL scores (β: 1.10, 95% CI: 0.40 to −1.8).

#### 3.1.4. The Contributory Factors Related to the Different Domains of Perceived Social Support (From Friends, Family, and Significant Other)

Model 2 (Table 5) showed that the different social support domains were positively associated with each other (family β: 0.45, 95% CI: 0.37–0.533, significant other β: 0.23, 95% CI: 0.16–0.30). A longer diagnostic interval (β: 0.0, 95% CI: 0.00 to −0.01), greater number of days in hospital during the first 12 months since diagnosis (β: 0.01, 95% CI: 0.00 to −0.02), poorer communication from the cancer specialist (β: 0.52, 95% CI: 0.01 to −1.03), the extent to which cancer affected participants’ lives (β: −0.123, 95% CI: −0.24 to −0.01), and how much participants experienced side effects from their cancer (β: 0.14, 95% CI: 0.03 to −0.25) were associated with greater social support from friends.

#### 3.1.5. The Contributory Factors Related to the Overall Perceived Impact of Cancer

Model 3 (Table 6) showed that a greater impact of cancer on participants’ lives was related to higher socioeconomic deprivation, reduced perceived control over cancer (β: −0.09, 95% CI: −0.14 to −0.04), a greater experience of side effects (β: 0.31, 95% CI: 0.25 to −0.37), greater concerns about cancer (β: 0.18, 95% CI: 0.11 to −0.25), and a greater emotional impact of cancer (β: 0.33, 95% CI: 0.26 to −0.39). Participants also reported a greater impact of cancer on their lives when they had spent a longer time in the hospital during the first 12 months (β: 0.01, 95% CI: 0.00 to −0.01), if they received surgery only (β: 3.69, 95% CI: 0.12 to −7.27), or if they received a transplant (β: 3.90, 95% CI: 0.19 to −7.61) (Table 6).

### 3.2. Patient Interpretation

Seven YAP members (5 female, 2 male) participated in the patient interpretation exercise. The YAP members were diagnosed with a range of cancer types common to AYAs ages 15–24 years. Their ages at diagnosis ranged from 15–27. At the time of the exercise, five were in stable relationships, two had children, and all were employed.

**Figure 1:**
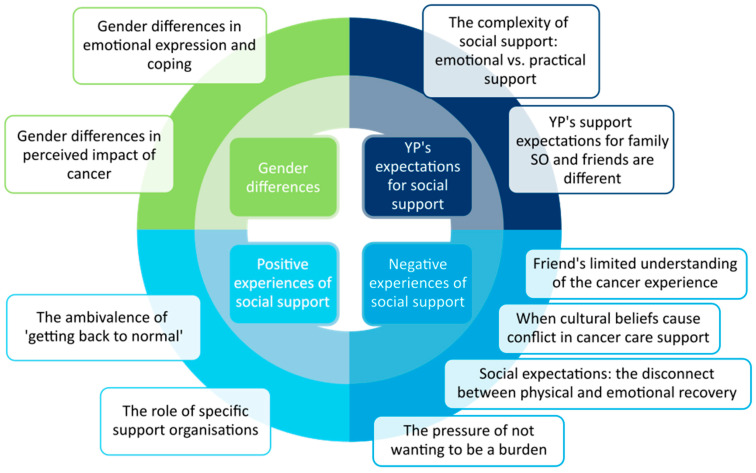
Themes from the patient interpretation (SO: significant other; YP: young person.).

Four cross-cutting themes related to the psychosocial experiences of AYAs with cancer were identified. These interpretations provided insight into gender differences, social support expectations, and both positive and negative experiences of social support, each with several constituent sub-themes (Figure 1).

#### 3.2.1. Gender Differences

Quantitative findings indicated that male participants consistently reported higher PSQOL scores across the study period compared to females. Insights from the YAP further elucidated this gender disparity. They suggested that males may adopt more emotionally detached coping strategies, often suppressing or downplaying their feelings, possibly as a reflection of societal expectations of male independence:

*“I didn’t really get seriously upset but I would disconnect. And that wasn’t me trying to put on a brave face. That was just what I did and obviously that isn’t to speak for males in general but rather than get really upset about something, I’d probably just watch videos or play a computer game or something like that. And just detach that way …maybe the male isn’t saying that they’ve had an absolutely terrible time. They’ve just been having a nothing time, because they’ve disconnected.”*

In contrast, female participants were perceived as more willing to articulate and process their emotions openly.

*“Is that because females are happier to explain their emotions over a guy that maybe didn’t want to say I was feeling quite upset at this moment in time, or I was lonely.”*

The YAP further reflected that this difference in emotional expression may also contribute to a heightened perception of cancer’s impact among females. Some female YAP members noted that hormonal fluctuations and societal pressures regarding appearance (e.g., concerns over scarring or hair loss) exacerbated their distress, thereby intensifying the perceived burden of cancer. For example, Maria (F) reflected:

*“* *I was focused on, I’ve just had surgery, I’ve got a big scar. What’s life going to be like, getting into relationships. I can’t wear certain outfits. Or how will I be able to get a job if I can’t climb stairs? Like little things that come into your head about side effects and dealing with them. Maybe for a guy they might just think oh, get my treatment, get better, get back to life.”*

#### 3.2.2. Expectations for Social Support

Quantitative analyses revealed that while support from friends was consistently rated higher than support from family or a significant other, it was the only social support domain with an association with PSQOL. The YAP highlighted distinct expectations for support depending on its source. They also emphasized that emotional and practical support were valued differently. Family members and significant others were typically expected to provide both tangible assistance (such as transportation and help with daily tasks) and emotional support, offering a more stable and familiar environment. In contrast, support from friends was viewed more variably. Friends, often not as intimately involved in the patient’s day-to-day challenges, were perceived as less capable of addressing the nuanced emotional needs of AYAs, as Steph (F) described:

*“Friends…don’t know you as well…Because they don’t see you as much, whereas my mum was there 24/7, boyfriend pretty much as well. They (friends) didn’t see everything. So didn’t always know where to…how to handle you, almost, or how to deal with you or how to act.”*

Instead, friends were valued more for their ability to re-establish a sense of normalcy or a pre-illness social identity. This divergence in support expectations lay at the core of participants’ positive and negative experiences of social support. When friends successfully reinforced normalcy by engaging in familiar activities and treating individuals as they would healthy peers, participants reported a positive influence on psychosocial wellbeing. Conversely, when friends fell short of these expectations (either by not fully understanding the emotional impact of cancer or by inadvertently emphasizing the disruptions caused by cancer), the YAP reported isolation and unmet emotional needs. These nuances are detailed in the following themes.

#### 3.2.3. Positive Experiences of Social Support

Quantitative data demonstrated that although greater perceived support from friends was linked to spending more time in the hospital, experiencing more side effects, and poorer PSQOL, it was also associated with a reduced overall impact of cancer on participant’s lives. The YAP described how positive social support experiences emerged when support contributed to a sense of normalcy. They recounted how their friends who treated them as they would treat any other peer helped to mitigate the isolating effects of cancer.

For example, Laura (F) shared that being treated like “*normal*” by friends allowed her to “*feel like a young person who could have a life*.”

Similarly, Lawrence (M) described how much he valued engaging in routine activities such as playing sports or casual outings with his friends:

*“The support I found most useful from my group of friends was actually probably … just when they really treated me normally and we’d play football at the park. And take the mickey out of me, rather than yes, when I was first diagnosed, people were a bit scared to do that and oh no, he must be really sad but I actually preferred the support from my friends being treat me like normal, play some sports or something.”*

Professional support organizations were also identified as valuable resources that provided tailored peer support and a community of understanding. These helped to further buffer against the disruptions caused by cancer, its treatment, and side effects. For example, Andrew (M) described how professionally organized peer support helped to bridge the gap in his support when he was first diagnosed with brain cancer, as his mother had received a cancer diagnosis shortly thereafter.

*“At the time it (support) was very little until I eventually found the Maggie’s centres, because they’re really good and then I could talk to people who I could relate to and who could offer support that I needed.”*

#### 3.2.4. Negative Experiences of Social Support

While the previous theme provides context for the positive aspects of social support in relation to reducing the overall impact of cancer, this relationship was multifaceted. The quantitative findings illustrated how increased social support from friends was also associated with poorer PSQOL. Although the YAP described how social connections with friends often helped maintain a sense of normalcy, these did not always meet their deeper emotional needs. For instance, while this support sometimes provided “*a good distraction*” (Laura, F) during hospital stays, it did not fully mitigate the emotional toll of prolonged hospitalization. In some cases, the same support that was intended to be uplifting contributed to feelings of isolation, emotional strain, and a disconnect between physical and psychological recovery. The YAP identified several negative dimensions of social support that spoke to the complexities of illness perceptions in the AYA cancer experience.

Firstly, the YAP explained how their friends often had a limited understanding of the complexities of cancer, and this often led to unmet emotional needs. For example, Laura (F) reflected:

*“None of us had really experienced anything too dramatic at that point. So it would have been really hard for them to know what to say. Sometimes they just didn’t want to talk about it, if I was bringing stuff up, it would be ‘oh no, but you’re going to be fine. So let’s move on.’”*

The YAP agreed that this largely related to the fact that often, they themselves did not know how to process or describe their own feelings, as Amy (F) reflected:

*“I think it’s very hard to be somebody with a friend that’s got cancer… there’s no understanding there. How can you understand something that’s so impactful to somebody and they don’t even understand themselves what they’re going through.”*

Moreover, participants reported a disconnect between their friends’ perceptions of their physical recovery and the ongoing emotional toll of cancer, with friends sometimes expecting a swift return to normalcy. This was particularly significant in those who continued to deal with the side effects of illness long after initial treatments, leading to frustration when friends could not understand the lingering impacts of fatigue, emotional distress, or physical limitations. For example, Amy (F) recounted:

*“When I actually had my operation, they (friends) were like ‘oh, you’ll be fine now, you’ll be good now. Oh, let’s go out. Let’s do this.’ And if I couldn’t go on a night out, it was like I was being an issue. So I then really tried my best to go and I’d go and then I’d be like ‘oh, it’s ten o’clock now and I am in agony. I need to go and lie down. I’m tired.’ ‘Oh come on, let’s just get another drink. Oh come on, don’t be so boring. Come on, let’s have a good night’. Thinking that I was just sad and needed to be made happy to stay out [laughs] and it wasn’t that. If they had a few shots of morphine or something then maybe I might have been fine. But that wasn’t the case.”*

This mismatch was further exacerbated by the pressure many participants felt to avoid burdening friends, leading some to withdraw despite having support available. For many, this reluctance stemmed from the emotional strain of maintaining a facade of wellbeing. Participants frequently described concealing their true feelings to protect others from additional stress. For example, Maria (F) reflected:

*“If anything with my friends, it was more a case of they wanted to come and see me but I was feeling guilty. I didn’t want them to feel like they would come and visit me and then I’m not feeling okay, so I’d have to pretend I’m feeling okay. I just don’t want that stress of having to pretend I’m okay. I just didn’t want to see them.”*

Several participants expressed guilt over imposing on friends or assumed the role of “*emotional managers*” to shield others from their distress—actions that ultimately reinforced feelings of isolation. For example, Amy (F) described:

*“I felt like a manager. I had to manage their feelings. I had to tell them it was going to be okay, to the point where I was playing down symptoms.”*

In some instances, cultural factors further complicated social interactions. Some participants described conflicts arising from cultural taboos within their family and wider communities that hindered open discussion about their illness, leaving them feeling isolated within their own communities. Maria (F) described how she “*Put up a barrier*” around her Nigerian friends:

*“For my being black and with my community, with the cancer situation, it’s quite a taboo to talk about. So although like I said I had quite a close family support with my mum and my siblings… I felt almost embarrassed and shy to talk about it with my friends… And again because nobody in our family and social support had ever gone through cancer, I was the only one in the Nigerian community at that time. So it was really awkward for me to even go out with my friends. It was like sometimes they didn’t know what to say or they didn’t know what to ask that wouldn’t come across as crossing a boundary of what’s the appropriate question to ask, or what’s appropriate not to say it. They were treading on eggshells a bit.”*

Similarly, Laura described how her immediate family members struggled to openly acknowledge or discuss her cancer due to cultural taboos surrounding health. Although she felt supported by her mother, she noted how her mother was not supported and the emotional strain this put on her family.

*“My parents are together but well he’s [father] about 20 years older than my mum. He’s Iranian and very traditional, very not understanding. So when I first got diagnosed I think he didn’t, even though he’s had lymphoma himself [laughs] but he didn’t seem to understand what I was going through. I don’t know if there was shame involved because it’s like ‘oh, my child is ill’. But yes, it was all very strange. He didn’t come to the hospital. He didn’t understand what was going on. He didn’t support my mum at all.”*

## 4. Discussion

### 4.1. Summary

Our study evidences the psychosocial needs of AYAs with cancer, drawing on theories of illness perception and social support. By contextualizing quantitative findings from a large, diverse longitudinal cohort with insights obtained through consultation with young people, our findings provide a nuanced perspective on how gender differences, social support dynamics, and illness perceptions collectively shape PSQOL in this population.

Across the 3 years post-diagnosis, females consistently reported lower PSQOL compared with males. Poorer PSQOL was also associated with negative illness perceptions, including greater emotional distress, a greater overall impact of cancer, heightened concerns about cancer, and a greater experience of side effects. In contrast, positive beliefs about treatment efficacy were related to higher PSQOL. Greater social support from friends was associated with poorer PSQOL; however, this relationship was complex. While greater social support from friends was associated with longer diagnostic intervals, hospital stays, and experiencing more cancer-related side effects, it was also linked to a reduced overall perceived impact of cancer.

Consulting with young people provided further context for these findings, revealing gender differences in emotional expression and the perceived impact of cancer; differing expectations for social support from friends versus family and significant others (with friends primarily valued for maintaining normalcy, while family and significant others were expected to provide both emotional and practical support); positive experiences of social support (particularly in maintaining a sense of normalcy and through professional support services); and negative experiences of social support, including friends’ limited understanding of cancer, the disconnect between physical and emotional recovery, the pressure to avoid burdening others, and cultural barriers that influenced social support dynamics.

Together, these findings imply a dual role in social support from friends as both a protective and potential risk factor in shaping illness perceptions and the psychosocial wellbeing of young people with cancer.

### 4.2. Comparison with Previous Literature and Implications for Future Research

#### 4.2.1. Gender Differences in PSQOL and Emotional Expression

The observed gender differences in PSQOL are consistent with existing literature indicating that female AYAs with cancer experience lower quality of life compared to males [52,53,54,55]. This disparity may partly be attributable to the influence of societal norms which shape emotional processing and coping strategies [56]. While both genders exhibit considerable overlap in their appraisal of stressors and emotional responses [57,58,59], empirical studies in adult cancer populations show that female patients typically experience heightened emotional processing and expression tendencies compared with males [60,61,62,63]. However, this responsiveness may intensify distress, particularly regarding body image and future relationships [64,65].

Our YAP consultation further contextualized these findings within the AYA population, suggesting that female AYAs may have felt a greater impact of cancer because of its effect on body image. This interpretation aligns with research indicating that female AYA cancer patients are more likely to experience a negative body image compared to males [66,67,68,69,70]. In particular, Zucchetti et al. reported that female AYA cancer survivors exhibit greater fears of weight gain and heightened worries about physical appearance compared to males [69]. The compounding effects of social pressures, body image dissatisfaction, and the psychosocial challenges of young adulthood suggest that female AYAs may require more targeted interventions that address these specific concerns. Considering the limited amount of quantitative data available on body image among AYA cancer patients, future studies could explore the longitudinal factors related to a negative body image within the context of cancer. These could provide valuable insight into the trajectory of body image concerns over time, helping to determine the most effective timing for interventions such as initiating discussions between health care professionals and AYAs [66,67]. Since many long-term effects of cancer can be mitigated through targeted surveillance and early intervention, healthcare professionals who provide multidisciplinary age-specific AYA care are well positioned to identify and address body image problems [67,69,71].

From a theoretical perspective, the gender differences in emotional expression implicated in our results resonate with the Transactional Model of Stress and Coping [30]. The model suggests that an individual’s stress response is influenced by their cognitive appraisal and coping mechanisms. Therefore, the male preference for emotional detachment may serve as a protective strategy, preventing overwhelming stress responses, whereas females’ greater emotional disclosure could contribute to increased self-awareness but also heightened distress. In adult cancer patients, emotional suppression is generally associated with poorer long-term psychological adjustment [72,73,74]. However, our results showed that male AYAs consistently reported higher PSQOL scores than females, suggesting that the relationship between emotional processing and PSQOL may differ between AYA and adult cancer populations. One possible explanation is that male AYAs may employ adaptive detachment or distraction-based coping strategies in the short-term [75,76], which may help them navigate the immediate psychosocial challenges of cancer without experiencing the same levels of distress as females. However, it is also possible that self-reported PSQOL measures do not fully capture the psychological needs of male AYAs. Future research into the psychosocial needs of AYAs with cancer would therefore benefit from direct exploration of gender-specific emotional processing strategies. Incorporating objective psychological assessments alongside qualitative methodologies could offer a more comprehensive understanding of potential unmet emotional needs.

#### 4.2.2. The Role of Culture in Shaping Illness Perceptions

Culture affects how individuals define and perceive disease as well as how they interpret, label, and explain symptoms [77,78]. Cultural factors play a crucial role in shaping the experience of illness and social support in the general population [25,79,80]. In many cultures, discussions about cancer are often taboo or associated with stigma, creating significant barriers to open communication about the illness [79,81,82]. Consultation with the YAP provided some preliminary evidence suggesting that this may be particularly true for AYAs with cancer. Anecdotal accounts indicated that cultural taboos around cancer lead to hesitancy in discussing cancer with peers and family members. Such reluctance was related to heightened isolation and complicated access to peer support during a vulnerable time.

These accounts align with broader findings that AYAs with cancer often suppress their emotions to avoid upsetting those around them or when cancer becomes a taboo topic within their social circles [83,84]. However, there is a lack of empirical evidence on how culture specifically influences illness perceptions and access to social support for AYAs with cancer. Although our YAP insights are valuable, our study lacked sufficient data to substantiate these findings. The BRIGHTLIGHT cohort comprised limited ethnic variation and did not directly capture information around cultural experience. Theoretically, cultural norms could have a profound influence on how AYAs perceive their illness and engage with social support systems. However, there is a significant evidence gap. Longitudinal studies that compare AYAs from different cultural groups could provide valuable insights into how cultural norms shape illness perceptions and psychosocial outcomes. By examining variations in attitudes toward cancer, communication about illness, and the availability and quality of social support across cultural contexts, researchers could identify culturally specific needs and challenges that influence PSQOL. Similarly, ethnographic techniques could explore the lived experiences of AYAs from diverse cultural backgrounds. This would help capture the nuanced ways in which cultural taboos and stigmas influence illness perceptions, coping strategies, and the availability and quality of social support networks. Researchers could specifically examine how AYAs navigate cultural barriers when discussing their cancer experience with peers, and how these factors impact their psychosocial wellbeing.

#### 4.2.3. The Role of Social Support in Shaping Illness Perceptions

The Illness Perception Model was developed as a focused extension of Leventhal’s Common-Sense Model (CSM [85]) to specifically examine cognitive and emotional representations of illness. It provides a useful framework for understanding the role of social support in shaping AYAs’ experiences of cancer. The model posits that individuals’ beliefs about their illness influence coping behaviours and psychosocial outcomes. Our quantitative findings indicate that the greater perceived impact of cancer was associated with poorer PSQOL. However, perceptions of the overall impact of cancer were reduced when participants perceived greater social support from friends, suggesting that peer interactions play a crucial role in shaping illness narratives.

These findings align with previous research highlighting that AYAs experience significant social identity disruptions post-diagnosis, particularly in their relationships with peers, coworkers, and close social network members [86]. Many AYAs perceive that cancer alters their existing social identities, leading to increased isolation, frustration, and difficulty maintaining pre-illness relationships [13,87]. Our YAP consultation demonstrated that support from friends sometimes reinforced a sense of normalcy, which appeared to buffer against negative illness perceptions [88]. This aligns with research suggesting that effective social support can enhance AYAs’ ability to integrate cancer into their identity, facilitating a process of “biographical reconstruction” where individuals work to reconcile their pre-cancer identity with their current illness experience [56,89,90].

However, the YAP highlighted that not all peer support is beneficial. When friends struggled to understand the complexities of cancer, social interactions often reinforced feelings of isolation and emotional distress. According to the Illness Perception Model, these negative experiences may arise because such interactions fail to validate AYAs’ lived experiences, reinforcing distress rather than alleviating it. The Illness Perception Model suggests that such interactions may contribute to identity conflicts and heightened uncertainty, particularly when support fails to align with AYAs’ evolving sense of self post-diagnosis. In the context of “biographical reconstruction”, effective social support from friends is contingent on how messages are communicated, the relational context, and the receiver’s interpretation [89,91]. AYAs may perceive well-intended but poorly framed support as invalidating, particularly when they emphasize resilience in ways that feel dismissive or reinforce feelings of dependency, stigma, or diminished self-worth [89]. Goldsmith et al. noted that while encouragement, empathy, and respect for autonomy are typically helpful, unrealistic expectations of resilience can be detrimental to AYAs’ adjustment to cancer [92,93].

#### 4.2.4. Cognitive Processing of Social Support

Cognitive-Behavioural Theory [94] would suggest that the psychosocial influence of social support from friends is not inherently positive or negative but rather depends on how individuals cognitively process their social interactions. When support reinforced a sense of normalcy, AYAs may have reframed their experiences in ways that lowered their perceived illness burden, despite ongoing physical challenges. Conversely, when support failed to address deeper emotional needs or imposed unrealistic expectations of resilience, it may have exacerbated maladaptive thought patterns and distress, perpetuating negative illness perceptions [95]. This interpretation also aligns with published research indicating that social support can facilitate “biographical reconstruction” when it allows AYAs to integrate their cancer experience into a coherent life narrative [56,89,90]. Cancer disrupts social identities, career plans, relationships, and normative life trajectories, requiring AYAs to reconstruct their biography in ways that integrate their illness experience into a revised sense of self [96,97].

#### 4.2.5. The Quality of Social Support from Friends

Consequently, the perceived quality of social support, rather than its mere presence, emerges as a key determinant of PSQOL. Social Support Theory posits that social support functions as a protective factor against stress but only when it aligns with individual needs [98]. The YAP revealed that AYAs placed distinct expectations on different social networks: family and significant others were expected to provide both emotional and practical support, while friends were primarily valued for maintaining normalcy. These expectations align with previous findings that distinguish between friends from before diagnosis and cancer peers. In their thematic analysis of AYA cancer patients’ experiences with friends and cancer friends, Kaluarachchi et al. demonstrated how the latter often offered a more nuanced understanding of the cancer experience, which may reinforce feelings of normalcy in ways that pre-existing friendships may not [99]. In our study, when friends failed to meet deeper emotional needs or imposed unrealistic expectations of coping, social support became a source of additional stress rather than relief. These findings align with research demonstrating that high levels of appropriate social support are associated with better coping, lower anxiety, improved decision-making, and enhanced quality of life for AYAs [86]. However, distorted relationships with social networks may impede effective support-seeking behaviours, complicating AYAs’ adjustment to illness and hindering biographical reconstruction [89,100].

#### 4.2.6. Gaps in Social Support Interventions

Most healthy AYAs have limited familiarity with cancer, so it is unsurprising that they may struggle to provide appropriate support to peers going through cancer. Despite this challenge, there is a notable gap in research evaluating social support interventions for AYAs with cancer and supporting those with cancer. Studies suggest that this population is highly engaged with both in-person and web-based social networks for seeking information and emotional support [101,102]. However, the YAP demonstrated that many young people only became aware of support and information services after prolonged suffering. This highlights a broader issue of barriers in accessing age-appropriate support services, with many AYAs underutilizing existing programs due to a lack of awareness or accessibility challenges [13].

Future research in this area should therefore explore the following:

1. The effectiveness of digital interventions, social media platforms, and structured peer-support programs in fostering meaningful peer relationships and enhancing social support for AYAs with cancer [103,104];
2. How social support interventions can be adapted to better align with AYAs’ expectations and normative social transitions, helping to support identity reconstruction [56];
3. The role of targeted interventions in equipping young people with communication strategies that help AYAs with cancer, ensuring that their support is perceived as helpful rather than distressing [92].

### 4.3. Strengths and Limitations

A key strength of this study is its integration of large longitudinal data with interpretive insights from patient consultation, offering a more nuanced understanding of illness perceptions, social support, and the psychosocial experiences of AYAs with cancer. However, common cohort study limitations such as selection bias, attrition, and missing data [105,106] may affect the generalizability and completeness of the findings. Similarly, although BRIGHTLIGHT recruitment spanned most English hospitals, the predominantly white sample narrows the applicability of the results to other ethnic groups and restricts exploration of culture, which is a key component of psychosocial experience [107,108,109]. Additionally, although the BRIGHTLIGHT dataset is one of the most comprehensive available for AYA cancer research, data collection occurred over a decade ago. Given advancements in medical and psychosocial care, findings may not fully reflect current healthcare contexts and AYA experiences [110]. Furthermore, the analyses in this study were confined to the outcome measures captured in the BRIGHTLIGHT survey, precluding the examination of additional psychosocial variables and confounders. For example, the database lacked cultural context, insights into emotional and cognitive processing, or the effects of information/support interventions.

Nevertheless, our analysis utilized well-established measures (BIPQ and MSPSS) which have been validated in AYA cancer research, affirming their suitability for assessing the intended constructs [111,112,113]. Similarly, use of DAG modelling helped to reduce “noise” in our model outputs from confounding variables. YAP engagement in the consultation exercise provided invaluable interpretations of the results, ensuring that patient perspectives were central to the interpretation of the results and addressing gaps in the existing literature. Although the YAP was self-selected, predominantly female (71%), and comprised individuals with patient involvement experience, their participation emphasized the importance of addressing the unique psychosocial needs of AYAs, tailoring medical and social care accordingly, and expanding research in this area. Notably, the YAP’s active and enthusiastic participation challenges the perception that AYAs are too vulnerable to participate in research or contribute meaningfully to service development and improvement [114,115].

## 5. Conclusions

This study advances understanding of the psychosocial experiences of AYAs with cancer by illustrating the unique psychosocial needs of AYAs compared to adult populations, particularly the pivotal role that peer support can play in preserving pre-illness identity and mitigating negative illness perception. However, social support from friends may also introduce challenges to individuals when expectations are unmet or when support interactions reinforce distress. Psychosocial interventions should therefore not only aim to increase peer support but prioritize enhancing the quality of social interactions to better meet the emotional and psychological needs of AYAs. Additional longitudinal research exploring the influence of emotional and cognitive processing as well as cultural and socioeconomic factors on social support experiences and PSQOL will further refine our understanding of these dynamics. Furthermore, studies that track long-term psychosocial outcomes beyond the 3 years post-diagnosis will be essential for understanding the sustained impact of social support across the cancer trajectory in AYAs.

## Figures and Tables

**Table 1:** Standardized questionnaires used for data collection from the BRIGHTLIGHT cohort.

Construct	Measure	Details
Perceived social support from friends, family, and significant other	The Multidimensional Scale of Perceived Social Support (MSPSS) [46]	Twelve statements, rated on a 7-point Likert scale. Support scores range from 4–28, with higher scores indicating more support.
Illness representation	The Brief Illness Perception Questionnaire (BIPQ) [47]	Emotional and cognitive representations of illness are measured using eight questions * with a fixed response scale specific for each question, for example, “not at all” to “extremely helpful”. Each question represents a single domain and therefore different dimensions of illness perception: consequence, personal control, treatment control, timeline, identity, coherence, emotional representation, and concern. Responses are scored 1–10; the higher the score, the greater perceived illness impact.
Psychosocial health-related quality of life (PSQOL)	The Paediatric Quality of Life Questionnaire (PedsQL) [48]	The full PedsQL contains 23 items rated using a 5-point Likert scale (never, almost never, sometimes, often, and almost always). Responses are presented as four domain scores (physical, emotional, social, and work/studies functioning), two summary scores (physical and psychosocial function), and a total score. Domain, summary, and total scores range from 0 to 100, with 100 representing the best possible QOL.

* The timeline domain was not included in the BRIGHTLIGHT Survey questionnaire; QOL: quality of life.

**Table 2:** Demographic characteristics.

	Male n = 457 (55%)	Female n = 373 (45%)	All n = 830
Age at diagnosis			
13–18 years	170 (37.2)	132 (35.4)	302 (36.4)
19–24 years	287 (62.8)	241 (64.6)	528 (63.6)
IMD Quintile			
1 (Most affluent)	96 (21)	88 (23.6)	184 (22.2)
2	74 (16.2)	62 (16.6)	136 (16.4)
3	92 (20.1)	64 (17.2)	156 (18.8)
4	97 (21.2)	85 (22.8)	182 (21.9)
5 (Least affluent)	91 (19.9)	67 (18)	158 (19)
Missing	7 (1.5)	7 (1.9)	14 (1.7)
Cancer severity			
Least	246 (53.8)	215 (57.6)	461 (55.5)
Intermediate	104 (22.8)	90 (24.1)	194 (23.4)
Most	107 (23.4)	68 (18.2)	175 (9.2)
Cancer type			
Haematological	195 (42.7)	178 (47.7)	373 (44.9)
Oncology	262 (57.3)	195 (52.3)	457 (55.1)
Long-term illness	29 (6.4)	47 (12.6)	76 (9.2)
Median diagnostic interval in days (SD)	58 (149.5)	87 (194.2)	62 (172.5)
Mean number of general practitioner (GP) visits before diagnosis (SD)			
	1.9 (2.6)	2.8 (4.2)	2.3 (3.4)

n (%); IMD: Index of Multiple Deprivation.

**Table 3:** Longitudinal changes in PSQOL, social support, and illness perception scores.

	Wave 1			Wave 2			Wave 3			Wave 4			Wave 5		
	Female	Male	All	Female	Male	All	Female	Male	All	Female	Male	All	Female	Male	All
Mean psychosocial QOL	65.41	75.36	70.89	70.69	80.07	75.93	71.42	80.54	76.40	71.75	81.58	77.07	74.22	83.02	79.28
Mean social support															
Friends	8.33	7.92	8.10	9.95	9.38	9.63	10.13	9.45	9.76	9.56	9.35	9.44	10.15	8.92	9.43
Family	6.39	6.27	6.32	8.17	7.74	7.92	8.36	8.02	8.18	8.61	8.18	8.37	8.47	8.28	8.36
Significant other	6.24	7.02	6.67	7.92	8.33	8.16	7.66	8.24	7.98	7.92	8.78	8.40	7.65	8.36	8.07
Mean illness perception *															
1. Consequences	6.69	5.94	6.28	5.66	4.62	5.07	5.51	4.01	4.69	5.40	3.82	4.50	4.72	3.66	4.10
2. Personal control	5.31	6.16	5.78	5.87	6.47	6.21	5.96	6.69	6.36	5.99	6.61	6.34	5.98	6.78	6.45
3. Treatment control	8.92	9.11	9.02	9.16	9.38	9.29	9.29	9.40	9.35	9.50	9.43	9.46	9.43	9.41	9.42
4. Identity	5.82	4.70	5.20	4.88	4.02	4.39	4.94	3.68	4.25	4.82	3.74	4.21	4.86	3.59	4.11
5. Coherence	5.82	4.70	5.20	4.88	4.02	4.39	4.94	3.68	4.25	4.82	3.74	4.21	4.86	3.59	4.11
6. Emotional representation	6.33	5.25	5.74	5.49	4.51	4.93	5.63	4.23	4.86	5.18	4.28	4.67	4.89	3.95	4.34
7. Concern	7.95	8.22	8.10	7.96	8.11	8.04	7.78	8.16	7.99	7.88	8.05	7.98	7.79	7.98	7.90

* 1: Consequences: how much does cancer affect your life; 2: personal control: perceived control over cancer; 3: treatment control: how much do you think your treatment has or will help; 4: identify: how much do you experience side effects from your cancer; 5: coherence: how well do you understand your cancer; 6: emotional representation: how much does your cancer affect you emotionally; 7: concern: how concerned are you about your cancer.

**Table 4:** Model 1: multivariable regression model predicting PSQOL *.

Psychosocial Quality of Life	Coefficient (95% Confidence Interval)	*p* Value
Gender	−3.96 (−6.62 to −1.29)	0.004
Illness perception		
Consequences	−0.94 (−1.35 to −0.53)	<0.001
Personal control	0.26 (−0.06 to 0.59)	0.107
Treatment control	1.10 (0.40 to 1.79)	0.002
Side effects	−0.75 (−1.14 to −0.37)	<0.001
Concern	−0.37 (−0.80 to 0.07)	0.098
Coherence	0.13 (−0.35 to 0.61)	0.591
Emotional representation	−1.56 (−2.02 to −1.11)	<0.001
Social support		
Family	0.23 (−0.08 to 0.53)	0.149
Friends	−0.77 (−1.01 to −0.54)	<0.001
Significant other	−0.01 (−0.27 to 0.25)	0.918
Communication With a cancer specialist	−0.52 (−2.36 to 1.31)	0.577
With nurse specialist	−0.63 (−2.73 to 1.46)	0.553
IMD quintile, 1 as reference group		
2	−2.26 (−6.39 to 1.87)	0.283
3	−2.26 (−6.20 to 1.68)	0.261
4	−1.34 (−5.26 to 2.57)	0.502
5	−1.22 (−5.25 to 2.81)	0.553
Disease severity (least as reference group)		
Intermediate	0.57 (−2.97 to 4.10)	0.754
Most	0.26 (−3.85 to 4.36)	0.902
Age at diagnosis	−0.23 (−0.65 to 0.19)	0.285
Long-term condition	−1.12 (−5.39 to 3.15)	0.606
Geographical location		
South West	2.58 (−2.94 to 8.10)	0.360
East of England	−1.53 (−8.87 to 5.82)	0.684
North West	−1.03 (−7.09 to 5.03)	0.739
Merseyside	2.37 (−5.65 to 10.39)	0.562
East Midlands	−1.08 (−6.09 to 3.93)	0.673
Yorkshire	0.40 (−4.46 to 5.27)	0.871
North East	−2.54 (−12.49 to 7.40)	0.616
Thames Valley	1.46 (−9.84 to 12.75)	0.800
London	2.82 (−1.30 to 6.94)	0.180
South Yorkshire	1.49 (−6.16 to 9.13)	0.703
Wessex	−2.75 (−8.49 to 2.98)	0.347
Treatment group		
SACT + RT	9.10 (−17.31 to 35.52)	0.499
SACT only	8.37 (−18.18 to 34.93)	0.537
Surgery + RT	14.18 (−12.46 to 40.82)	0.297
Surgery + SACT	12.46 (−13.83 to 38.74)	0.353
Surgery only	12.23 (−14.03 to 38.50)	0.361
Surgery + RT + SACT	10.74 (−15.56 to 37.04)	0.424
Transplant	0.64 (−26.98 to 28.27)	0.964
Cancer type	−3.42 (−9.10 to 2.25)	0.237
Ethnic group	0.16 (−3.83 to 4.15)	0.938
Diagnostic interval	−0.01 (−0.02 to −0.00)	0.047
Number of GP visits	0.12 (−0.30 to 0.55)	0.575
Total days in hospital at 12 months post-diagnosis	−0.03 (−0.07 to 0.00)	0.068

* Minimal adjustment set: age, cancer severity, cancer type, HCP communication (cancer specialist + nurse specialist), ethnicity, gender, geography, hospital duration, long-term condition, socioeconomic status (IMD), treatment group. GP: general practitioner; IMD: Index of Multiple Deprivation; RT: radiotherapy; SACT: systemic anti-cancer treatment.

**Table 5:** Model 2: predictors of social support from friends *.

Social Support from Friends	Coefficient	95% CI		*p* Value
Gender	−0.19	−1.37	to 0.98	0.746
Age group at diagnosis	−0.74	−1.81	to 0.33	0.174
Age × gender	0.49	−0.98	to 1.96	0.511
Illness perception				
Consequences	−0.123	−0.24	to −0.01	0.039
Personal control	−0.03	−0.12	to 0.07	0.574
Treatment control	0.09	−0.11	to 0.29	0.375
Side effects	0.14	0.03	to 0.25	0.012
Concern	0.09	−0.04	to 0.21	0.176
Coherence	−0.02	−0.16	to 0.12	0.739
Emotional representation	0.01	−0.12	to 0.14	0.867
Social support				
Family	0.45	0.37	to 0.53	<0.001
Significant other	0.23	0.16	to 0.30	<0.001
Communication With cancer specialist	0.52	0.01	to 1.03	0.046
With nurse specialist	0.28	−0.30	to 0.87	0.339
IMD quintile, (1 as reference group)				
2	0.23	−0.92	to 1.39	0.692
3	−0.26	−1.36	to 0.84	0.639
4	0.14	−0.96	to 1.23	0.807
5	0.28	−0.84	to 1.40	0.624
Disease severity (least as reference group)				
Intermediate	−0.73	−1.72	to 0.25	0.144
Most	−0.72	−1.86	to 0.43	0.22
Long-term condition	1.129341	−0.07	to 2.33	0.065
Geographical locations				
South West	−0.08	−1.61	to 1.46	0.922
East of England	−1.40	−3.44	to 0.64	0.18
North West	0.99	−0.69	to 2.68	0.249
Merseyside	0.74	−1.50	to 2.98	0.517
East Midlands	−0.51	−1.92	to 0.90	0.476
Yorkshire	0.06	−1.29	to 1.42	0.928
North East	−0.54	−3.30	to 2.22	0.702
Thames Valley	0.24	−2.94	to 3.41	0.884
London	0.62	−0.53	to 1.77	0.29
South Yorkshire	−0.15	−2.29	to 1.99	0.891
Wessex	0.35	−1.25	to 1.95	0.668
Treatment group				
SACT + RT	0.05	−7.56	to 7.66	0.989
SACT only	−1.06	−8.73	to 6.58	0.783
Surgery + RT	0.30	−7.36	to 7.97	0.94
Surgery + SACT	−0.66	−8.24	to 6.92	0.86
Surgery only	−0.02	−7.59	to 7.55	1.00
Surgery + RT + SACT	−0.44	−8.03	to 7.14	0.91
Transplant	1.13	−6.81	to 9.08	0.78
Cancer type	−0.78	−2.37	to 0.81	0.34
Ethnic group	0.26	−0.85	to 1.38	0.64
Diagnostic interval	0.00	0.00	to 0.00	0.03
Number of GP visits	−0.03	−0.15	to 0.09	0.65
Total days in hospital at 12 months post-diagnosis	0.01	0.00	to 0.02	0.02

* Minimal adjustment set: age, cancer severity, cancer type, HCP communication (cancer specialist + nurse specialist), ethnicity, gender, geography, hospital duration, long-term condition, socioeconomic status (IMD), treatment group. GP: general practitioner; IMD: Index of Multiple Deprivation; RT: radiotherapy; SACT: systemic anti-cancer treatment.

**Table 6:** Model 3: predictors of the overall impact of cancer on young people’s lives (illness perceptions) *.

Illness Perception (Consequences)	Coefficient	95% CI		*p* Value
Gender	−0.16	−0.64	0.32	0.506
Age group at diagnosis	−0.04	−0.48	0.40	0.865
Age × gender	−0.02	−0.62	0.59	0.959
Illness perception				
Personal control	−0.09	−0.14	−0.05	<0.001
Treatment control	−0.06	−0.17	0.05	0.290
Side effects	0.31	0.25	0.37	<0.001
Concern	0.18	0.12	0.25	<0.001
Coherence	−0.07	−0.14	0.00	0.052
Emotional representation	0.33	0.26	0.39	<0.001
Social support				
Friends	−0.04	−0.07	−0.01	0.022
Family	−0.01	−0.06	0.04	0.651
Significant other	−0.00	−0.04	0.04	0.888
Communication With cancer specialist	0.04	−0.17	0.25	0.733
With nurse specialist	0.17	−0.07	0.41	0.158
IMD quintile, (1 as reference group)				
2	0.56	0.08	1.04	0.023
3	0.53	0.08	0.99	0.022
4	0.58	0.13	1.03	0.012
5	0.30	−0.17	0.76	0.215
Disease severity (least as reference group)				
Intermediate	0.09	−0.32	0.50	0.661
Most	0.25	−0.22	0.72	0.3
Long-term condition	0.03	−0.47	0.52	0.92
Geographical location				
South West	0.16	−0.47	0.79	0.616
East of England	−0.38	−1.20	0.43	0.358
North West	0.13	−0.57	0.84	0.711
Merseyside	0.79	−0.15	1.72	0.099
East Midlands	0.36	−0.22	0.94	0.226
Yorkshire	0.46	−0.10	1.01	0.104
North East	0.54	−0.60	1.68	0.352
Thames Valley	0.09	−1.21	1.40	0.888
London	0.41	−0.05	0.89	0.083
South Yorkshire	0.57	−0.31	1.45	0.203
Wessex	−0.01	−0.67	0.65	0.98
Treatment group				
SACT + RT	3.25	−0.34	6.84	0.076
SACT only	2.99	−0.62	6.59	0.105
Surgery + RT	3.51	−0.10	7.12	0.057
Surgery + SACT	3.31	−0.26	6.89	0.069
Surgery only	3.69	0.12	7.27	0.043
Surgery + RT + SACT	3.26	−0.32	6.83	0.074
Transplant	3.90	0.19	7.61	0.039
Cancer type	−0.45	−1.12	0.22	0.187
Ethnic group	−0.13	−0.59	0.33	0.588
Diagnostic interval	0.00	−0.00	0.00	0.838
Number of GP visits	0.01	−0.04	0.06	0.618
Total days in hospital at 12 months post-diagnosis	0.01	0.00	0.01	0.002

* Minimal adjustment set: age, cancer severity, cancer type, HCP communication (cancer specialist + nurse specialist), ethnicity, gender, geography, hospital duration, long-term condition, socioeconomic status (IMD), treatment group. GP: general practitioner; IMD: Index of Multiple Deprivation; RT: radiotherapy; SACT: systemic anti-cancer treatment.

## Data Availability

We welcome collaboration; for general data sharing enquiries, please contact RMT (rtaylor13@nhs.net).

## Supplementary Materials

The following supporting information can be downloaded at: https://www.mdpi.com/article/10.3390/cancers17121918/s1, Figure S1: Causal Diagram; Figure S2: Key findings from Phase 1 analysis. Table S1: Discussion guide: social support and gender differences in PSQOL. Table S2: Model optimisation.
