# Supplementary material for: Longitudinal Cohort Study of the Relationship Between Illness Perception, Perceived Social Support, and Psychosocial Quality of Life in Adolescents and Young Adults Newly Diagnosed with Cancer: Outcomes from a BRIGHTLIGHT Study
Source: Cancers (Basel). 2025 Jun 9;17(12):1918. doi: 10.3390/cancers17121918 (PMC12190661; doi:10.3390/cancers17121918)
Supplement: Supplementary file 1 [file cancers-17-01918-s001.zip › cancers-3635072-supplementary.pdf]

Supplemental Files

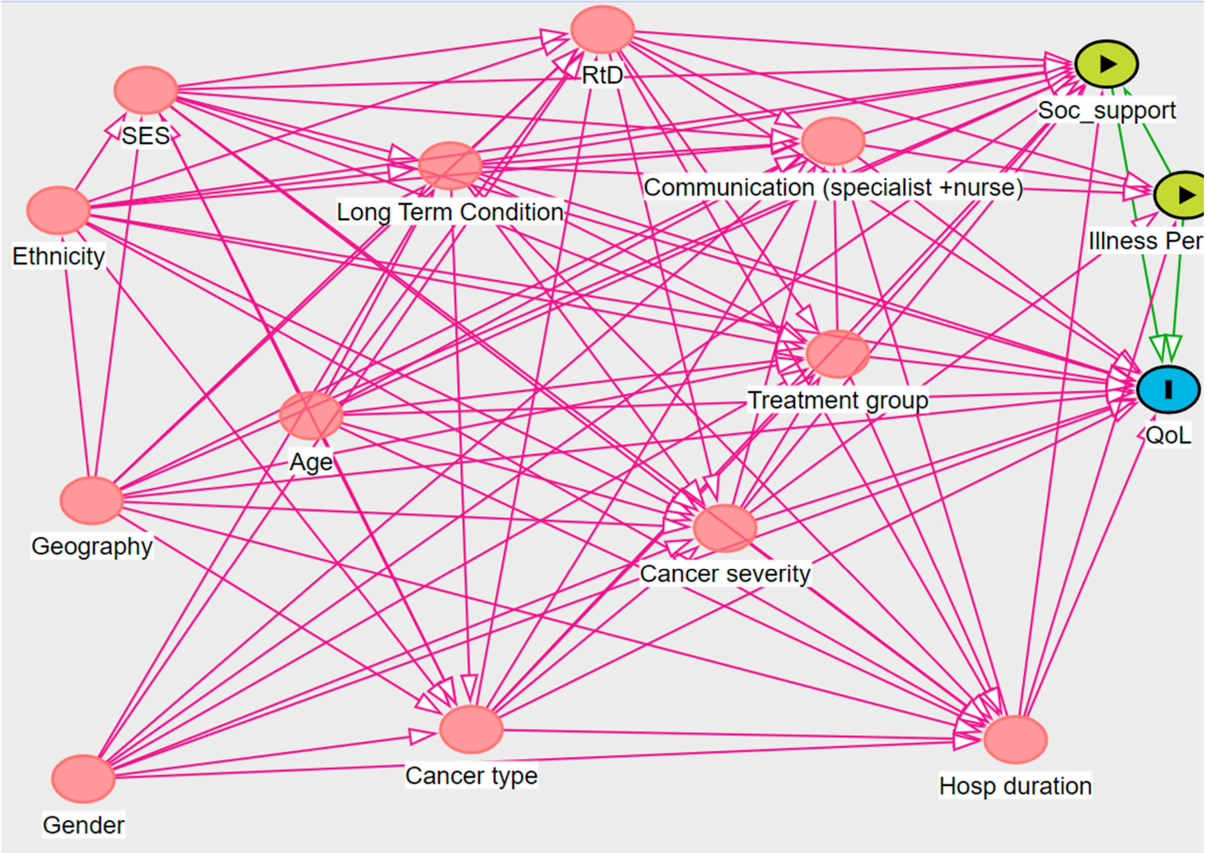

Figure S11: Causal Diagram

- Social support from **friends** was related to **poorer** quality of life. Social support from family or a significant other was not related in any way to quality of life.

- **Greater** social support from friends was related to:
  - ↑ **more time** spent in hospital
  - ↑ Feeling a **greater impact of cancer** in general
  - ↑ experiencing **more side effects** from cancer
  - ↓ **poorer** communication from the cancer specialist

- Some feelings about cancer (like how much it affected participant's lives in general, emotionally, and in terms of side effects) were related to **poorer** quality of life

- Participants who thought that their treatment could, or had helped experienced **better** quality of life

- Quality of life was generally **lower** in people identifying as **female** and in people who spent **more time in hospital**

Figure S2: Key findings from Phase 1 analysis

Table 1S1: Discussion guide: social support and gender differences in PSQOL

	Family	Friends	Significant Other
What types of support were most helpful to you?			
Were there times when support wasn't helpful, or made things harder? Why do you think that happened?			
What would have made that support more helpful?			
How did the support you received help you specifically while you were in the hospital?			
Was the support you received helpful in communicating with or understanding your cancer specialist?			
How did the support you received affect how much cancer affected your life?			
How did the support you received impact how your cancer affected you emotionally?			
How did the support you received impact your ability to manage or cope with side effects of your cancer?			
How have your relationships changed since your diagnosis?			
Quality of life was generally lower in people identifying as female[illegible] What do you think the male/female the differences are related to?			

Table S2: Model optimisation

Model	BIC
1 Psychosocial PEDS mixed model	7070.82
Psychosocial PEDS mixed model with interaction terms for wave and gender	7099.256
2 Social support mixed model	4850.172
Social support mixed model with interaction terms for wave and gender	4622.649
3 Illness perception mixed model	3795.834
Illness perception mixed model with interaction terms for wave and gender	3802.604
